# Application of low-order potential solutions to higher-order vertical traction boundary problems in an elastic half-space

**DOI:** 10.1098/rsos.180203

**Published:** 2018-05-09

**Authors:** Adam G. Taylor, Jae H. Chung

**Affiliations:** Computer Laboratory for Granular Physics Studies, Geosystems Engineering, University of Florida, FL, USA

**Keywords:** Boussinesq's problem, potential theory, soil–structure interaction, shallow foundation, elastic settlement analysis

## Abstract

New solutions of potential functions for the bilinear vertical traction boundary condition are derived and presented. The discretization and interpolation of higher-order tractions and the superposition of the bilinear solutions provide a method of forming approximate and continuous solutions for the equilibrium state of a homogeneous and isotropic elastic half-space subjected to arbitrary normal surface tractions. Past experimental measurements of contact pressure distributions in granular media are reviewed in conjunction with the application of the proposed solution method to analysis of elastic settlement in shallow foundations. A numerical example is presented for an empirical ‘saddle-shaped’ traction distribution at the contact interface between a rigid square footing and a supporting soil medium. Non-dimensional soil resistance is computed as the reciprocal of normalized surface displacements under this empirical traction boundary condition, and the resulting internal stresses are compared to classical solutions to uniform traction boundary conditions.

## 1. Introduction

The classical solution to the problem of a semi-infinite homogeneous, elastic body subjected to vertical loads on its boundary surface was first developed by Boussinesq [1] and discussed in more depth by Love [2]. The solution is derived from the Green's function of the Laplace equation, which was used to determine the stress equilibrium within an elastic half-space. Other solutions can be also obtained using a separate approach involving Bessel functions [3,4]. The technique involving potentials was concurrently applied in line with the theory of elastic contact proposed by Hertz [5]. Further, Newmark [6] provided a concise closed-form solution to the distribution of vertical stress at the corner of a uniform rectangular load. A general solution to this type of boundary-value problem was presented earlier by Love [7], who developed expressions for displacement and stress under any integrable distributions of vertical tractions over an arbitrary domain. Closed-form solutions to uniform tractions over a rectangular area were explicitly presented in his paper (with the exception of those involving vertical displacement). Only partial solutions were provided for surface tractions that linearly and bilinearly vary in a rectilinear domain.

Numerous attempts have been made to complete the integral calculations presented in Love's original work and to further expand the application of his closed-form solutions. Notably, Ahlvin & Ulery [8] developed tables for stress, strain and displacement under a uniform circular load from Love's equations, but they did not produce new closed-form solutions of potential functions. Schmertmann [9] proposed a semi-empirical strain influence method that has been widely used for elastic settlement analysis of shallow foundations based on the assumption that traction fields are uniform at the contact interface between a rigid footing and supporting soils. Recently developed closed-form solutions of the potential functions include those reported by Becker & Bevis [10], who completed Love's discussion of displacement under a uniform rectangular load. Dydo & Busby [11] further discussed linear and bilinear variations in vertical traction fields over a rectangular contact domain and provided one of the most comprehensive sets of closed-form solutions of the potential functions to date. However, the derivatives required to calculate stress, strain and displacement were omitted from their results. Li & Berger [12] developed a corresponding set of closed-form solutions for constant, linear and bilinear tractions over triangular domains. Most recently, Marmo & Rosati [13] suggested a general solution to a problem of polynomial surface-traction conditions over areas defined by arbitrary polygons. Importantly, Kunert [14] developed closed-form solutions for Hertzian-like contact pressures varying over a rectangular area.

Of all the historical contributions to this problem, Boussinesq's point-load and Newmark's uniform surface-load solutions stand out as engineering solutions to foundation design problems. These stress-influence methods are widely accepted in foundation design practice, but Love's closed-form solutions have rarely been viewed as practical design tools. It is laborious to solve the integral equations of the potentials in closed form even for the simplest of polynomial traction boundary conditions. Further, contact traction fields relevant to modern engineering applications may not be of such a low order. As it stands, a new set of closed-form solutions must be developed for higher-order boundary conditions, for which in most cases the calculations are intractable. Alternatively, a numerical model could be designed to obtain an approximate solution to a desired degree of accuracy specific to a prescribed boundary condition, but such a modelling effort can be time-consuming and very costly in iterative design processes.

It is, however, possible to balance mathematical rigorousness and computational efficiency in an alternative solution. A high-order surface traction field can be discretized in the loaded domain using piecewise approximation of lower-order polynomials. Very recently, work has been done to solve elastic contact problems by the superposition of solutions to linear tractions over triangular regions [15]. This technique is analogous to the collocation method used in the indirect boundary element method [16,17]. The lower-order solutions for each discretized subdomain are superimposed to approximate the displacement and stress fields in the elastic body (i.e. the principle of superposition). However, in the present study, it is proposed to calculate the ‘boundary integrals’ analytically for rectangular regions. This way, the superposition of low-order closed-form solutions replaces costly numerical quadrature. This solution approach is not entirely new [18,19]. It has been used to model the micro-contact of rough surfaces using Love's constant load solutions [20]. However, superimposing solutions to only the constant and unidimensional linear boundary conditions cannot lead to a continuous expression across the boundaries of rectangular subdomains. Consequentially, singularities or discontinuities will appear in the resulting stress fields within the loaded domain. The missing component for a robust solution approach to a higher-order traction boundary-value problem using rectangular regions appears to be the closed-form solutions of the bilinear (hyperbolic–paraboloidal) potentials. The authors have not found a complete set of solutions of the bilinear potentials in the literature. The goals of this study are to close this gap in the literature and to provide an approximate yet general solution approach to the Boussinesq–Love problem for higher-order traction boundary conditions.

In this paper, a finite number of closed-form solutions for lower-order boundary conditions are superimposed to accurately approximate a solution for high-order surface traction fields acting on a rectangular surface area of a homogeneous, elastic half-space. We develop a complete set of closed-form solutions for the bilinear hyperbolic–paraboloidal potential functions and their required derivatives. The potentials corresponding to the bilinear interpolants fitting the values of the four corners of each of a given number of discretized subdomains are superimposed, providing an approximation of the potential for the prescribed boundary condition and ensuring continuity across local boundaries. For foundation analysis applications, a surface traction function is empirically formulated by curve fitting pointwise contact-pressure data from past foundation experiments in the literature. This is then prescribed as a boundary condition for the half-space problem. We solve for select displacements, stresses and strains that occur in the body. The predicted distribution of non-dimensionalized vertical resistances (i.e. contact stiffness) is calculated as the reciprocal of normalized vertical displacements in the contact plane. A unique resistance distribution results directly from the corresponding non-uniform vertical boundary traction, which appears to evolve with increased applied load [21,22].

## 2. Governing equations and boundary conditions

**Figure 1.**
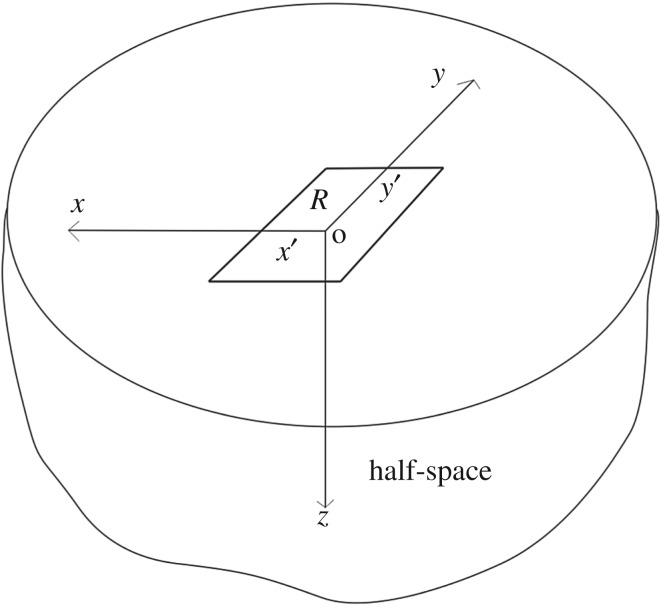
A description of the coordinate system and geometry of a rectangular loaded domain on the planar surface of a semi-infinite half-space.

A set of right-handed Cartesian coordinates is defined so as to describe an elastic body as half-space bounded by a plane at *z*=0, where the positive *z*-axis points downwards into the body. Let (*x*,*y*,*z*) be an arbitrary point within the body or on the boundary, while (*x*′,*y*′) designates a location within the region of contact *R* as shown in figure 1.

The stress equilibrium of a homogeneous, isotropic and elastic body can be stated as a system of partial differential equations in terms of the displacements (*u*,*v*,*w*) in the directions (*x*,*y*,*z*) as follows [2]:

2.1 $$\begin{array}{rl} & \;(\lambda +\mu )\frac{\partial D}{\partial x}+\mu \Delta u=0,\\ & \;(\lambda +\mu )\frac{\partial D}{\partial y}+\mu \Delta v=0\\ \text{and}\; & \;(\lambda +\mu )\frac{\partial D}{\partial z}+\mu \Delta w=0, \end{array}\}$$

where *D*=∂*u*/∂*x*+∂*v*/∂*y*+∂*w*/∂*z* is the strain dilation; *λ* and *μ* are the Lame's constants; and Δ is the Laplacian differential operator with respect to the spatial coordinates.

This problem reduces to a problem of potential theory when either surface displacements or tractions are given. This is achieved by means of Green's theorem and Betti's reciprocal theorem (see Love's treatise [2], Ch. X for details). The equilibrium state of the half-space is then given in terms of functions which satisfy the Laplace equation:

2.2 ΔV=0.

For a comprehensive description of complete solutions in terms of potentials, see §44 of reference [23]. The form of the potentials used to satisfy equation (2.1) is determined uniquely by the given boundary values.

The boundary conditions are defined as the values of surface displacements or tractions, corresponding to Dirichlet and Neumann conditions for this solution, respectively. It may be reasonable to model the mutual interaction of the foundation and continuum as a mixed boundary value problem, as is required for rigid punch problems [24,25] and Hertz–Mindlin contact theories [5,26]. Solutions to problems regarding this class of boundary conditions are mathematically cumbersome, although work has been done to develop a general solution [27]. If the footing is assumed to be flat, rigid and symmetrically loaded normal to the contact plane, vertical surface displacement *w*(*x*′,*y*′) can be assumed to be uniform within the area of contact (i.e. a constant displacement (Dirichlet) condition on *R*). Subsequently, the lack of contact outside of *R* corresponds to a zero vertical traction, *p*, a Neumann condition elsewhere on the boundary. The boundary conditions for equation (2.2) under these assumptions are written as follows:

2.3 $$\begin{array}{rl} & {w|}_{z=0}=\text{constant for }(x,y)\in R\\ \text{and}\; & {p|}_{z=0}=0\;(x,y)\notin R. \end{array}\}$$

The solution to this boundary value problem yields a vertical stress distribution with an absolute minimum at the centre and infinite stresses along the edges of *R* [24,25]. The singular values in the stress distribution are non-physical, and the material cannot be in equilibrium.

By contrast, the simplicity and convenience of the stress-influence methods stem from an assumption of a unique distribution of vertical traction prescribed over the area of contact. The resulting Neumann boundary condition can be expressed as

2.4 $${\frac{1}{2\pi }\;\frac{\partial V}{\partial z}|}_{z=0}=\left\{ \begin{array}{ll} p(x^{\prime },y^{\prime }) & \text{for }(x,y)\in R,\\ 0 & \text{for }(x,y)\notin R. \end{array}\right.$$

The resulting displacement and stress fields satisfy equation (2.1), are continuous within the body and lack singularities, providing that the surface tractions *p*(*x*′,*y*′) are bounded and uniformly equal to zero on the boundary of *R* [7]. Our following discussion will be focused on the application of potentials to solving this particular class of boundary value problems.

## 3. Potential functions for arbitrary contact pressure distributions

**Figure 2.**
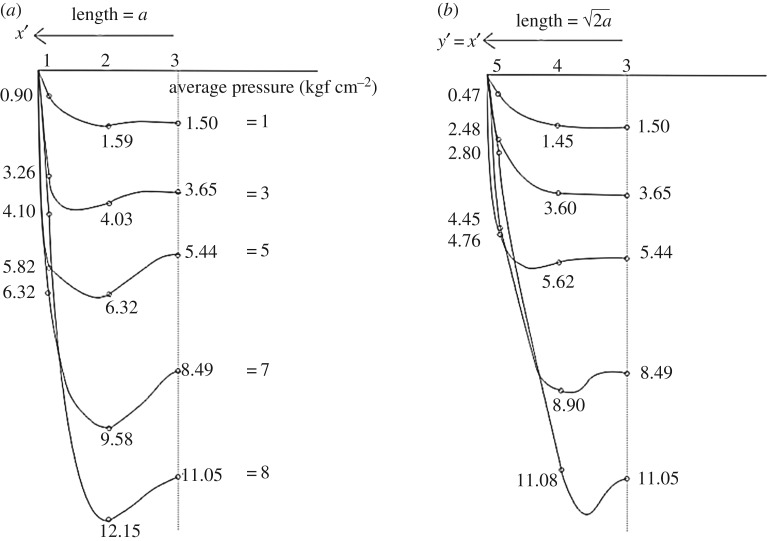
(Not scaled) Pressure variation for five loading cases extrapolated from data measured by Murzenko [21] at points (*a*) across the centre of a square footing, and (*b*) across its diagonal. This behaviour is assumed to be symmetrical across the rest of the footing, outlining a two-dimensional pressure surface.

Numerous empirical measurements have shown that the distribution of stress between a rigid structure and granular soil has relatively high-order spatial variation. Examples include Terzaghi's model [28], which is associated with general shear failure modes of shallow foundations in which a roughly parabolic distribution of contact pressure may develop. The laboratory test results reported by Bauer *et al*. [29] also show approximate parabolic pressure distributions on a scaled rectangular footing under an assumption of plane strain conditions. Murzenko [21] presents a set of contact pressure distributions that appear to be saddle-shaped (or shaped like the back of a two-humped camel) and correspond to pressure peaks occurring at a certain distance from the centre of the contact plane, a dip at the centre, and zero values along the edges, as shown in figure 2. Further, the contact pressure peaks observed in his experiments tend to move inwards towards the centre of the footing as applied load increases. This phenomenon has been reported in the results of a number of analytical models. Smoltczyk [22] presented an analytical expression for pressure boundary conditions mimicking this behaviour via statistical analysis, while Kerr [30] produced the same expression by introducing a shear membrane and another layer of springs into a Winkler-type [31] model. Furthermore, a number of pressure distributions with these attributes have been derived using elastoplasticity [32–34]. Interestingly, these phenomenological observations and analytical predictions show remarkable resemblance to the normal stress distributions measured empirically in sandpile models [35–38] and corresponding analytical models [39–42].

Considering these various contact pressure distributions in conjunction with the Neumann problem in equation (2.4), we formulate a general method of obtaining approximate solutions for any given surface traction while retaining the continuity of the displacement and stress fields within the body and upon its boundary. The simplest method of continuously approximating an arbitrary surface traction over a rectangular area is bilinear interpolation. Just as a two-dimensional boundary condition can be approximated to any degree of accuracy by a piecewise bilinear function, a solution specific to equation (2.4) can be accurately approximated by the superposition of each solution of the subdomain to a discretized Neumann boundary condition. These in turn are constructed as linear combinations of closed-form solutions for constant, linear and bilinear tractions, which are presented in appendix B.

Consider a given normal pressure *p*(*x*′,*y*′) on a rectangular region *R*={(*x*′,*y*′,0)|*a*_2_≤*x*′≤*a*_1_,*b*_2_≤*y*′≤*b*_1_} on the surface of a half-space. The distribution of pressure can be defined piecewise by a combination of any arbitrary functions. Using the coordinate system defined in figure 1, the distance between an arbitrary point in the body and a point on the surface within the loaded region is given by

3.1 $$r=\sqrt{{\left(x^{\prime }-x\right)}^{2}+{\left(y^{\prime }-y\right)}^{2}+z^{2}}.$$

As tractions normal to the surface boundary are assumed to be zero outside the region of contact, the potential function can be written as a double integral over the geometry of the region *R*:

3.2 $$V=\int_{b_{2}}^{b_{1}}\int_{a_{2}}^{a_{1}}\frac{p(x^{\prime },y^{\prime })}{r}\;dx^{\prime }\;dy^{\prime }.$$

This is the general solution to the Laplace equation subjected to the Neumann boundary condition in the half-space. To complete the equilibrium solutions of equation (2.1), it is necessary to introduce Boussinesq's logarithmic potential [1,2]:

3.3 $$\chi =\int_{b_{2}}^{b_{1}}\int_{a_{2}}^{a_{1}}p(x^{\prime },y^{\prime })\log(z+r)\;dx^{\prime }\;dy^{\prime }.$$

This function is related to equation (3.2) by *V* =∂*χ*/∂*z*, and both satisfy the Laplace equation (i.e. Δ*V* =Δ*χ*=0). The complete three-dimensional displacement, stress and strain fields are determined by these two potential functions and their derivatives, as shown in appendix A.

### 3.1. Superposition of potentials

Let us divide the loaded area *R* into *M* and *N* uniform intervals in the directions of *x*′ and *y*′ , respectively, which creates *M*×*N* disjoint rectangular subdomains:

3.4 $$R_{ij}=\{(x^{\prime },y^{\prime },0)|x_{i}\leq x^{\prime }\leq x_{i+1},y_{j}\leq y^{\prime }\leq y_{j+1}\}$$

for *i*=1,2,…,*M*, and *j*=1,2,…,*N*, such that $R=⋃R_{ij}$. Each individual subdomain has a length of Δ*x*=|*a*_1_−*a*_2_|/*M* and width of Δ*y*=|*b*_1_−*b*_2_|/*N*; the grid points at the corners of the subdomains are given by *x*_*k*_=*a*_2_+(*k*−1)Δ*x*, and *y*_*l*_=*b*_2_+(*l*−1)Δ*y* for *k*=1,2,…,*M*+1 and *l*=1,2,…,*N*+1.

Bilinear interpolation of the values of *p*(*x*′,*y*′) is performed at the four corners of each *R*_*ij*_, with the following system of equations:

$$\begin{array}{rl} p(x{{\;}^{\prime }}_{i},y{{\;}^{\prime }}_{j}) & \;=c_{ij}^{00}+c_{ij}^{10}x{{\;}^{\prime }}_{i}+c_{ij}^{01}y{{\;}^{\prime }}_{j}+c_{ij}^{11}x{{\;}^{\prime }}_{i}y{{\;}^{\prime }}_{j},\\ p(x{{\;}^{\prime }}_{i},y{{\;}^{\prime }}_{j+1}) & \;=c_{ij}^{00}+c_{ij}^{10}x{{\;}^{\prime }}_{i}+c_{ij}^{01}y{{\;}^{\prime }}_{j+1}+c_{ij}^{11}x{{\;}^{\prime }}_{i}y{{\;}^{\prime }}_{j+1},\\ p(x{{\;}^{\prime }}_{i+1},y{{\;}^{\prime }}_{j}) & \;=c_{ij}^{00}+c_{ij}^{10}x{{\;}^{\prime }}_{i+1}+c_{ij}^{01}y{{\;}^{\prime }}_{j}+c_{ij}^{11}x{{\;}^{\prime }}_{i+1}y{{\;}^{\prime }}_{j}\\ \mathrm{and}\;p(x{{\;}^{\prime }}_{i+1},y{{\;}^{\prime }}_{j+1}) & \;=c_{ij}^{00}+c_{ij}^{10}x{{\;}^{\prime }}_{i+1}+c_{ij}^{01}y{{\;}^{\prime }}_{j+1}+c_{ij}^{11}x{{\;}^{\prime }}_{i+1}y{{\;}^{\prime }}_{j+1}. \end{array}$$

Solving for the coefficients $c_{ij}^{mn}$ of Lagrange polynomials of first order per superscript indices *m*=0,1 for *x*′ and *n*=0,1 for *y*′, we have the following:

$$\begin{array}{rl} c_{ij}^{00} & \;=\frac{x{{\;}^{\prime }}_{i+1}y{{\;}^{\prime }}_{j+1}p(x{{\;}^{\prime }}_{i},y{{\;}^{\prime }}_{j})-x{{\;}^{\prime }}_{i+1}y{{\;}^{\prime }}_{j}p(x{{\;}^{\prime }}_{i},y{{\;}^{\prime }}_{j+1})-x{{\;}^{\prime }}_{i}y{{\;}^{\prime }}_{j+1}p(x{{\;}^{\prime }}_{i+1},y{{\;}^{\prime }}_{j})+x{{\;}^{\prime }}_{i}y{{\;}^{\prime }}_{j}p(x{{\;}^{\prime }}_{i+1},y{{\;}^{\prime }}_{j+1})}{\Delta x\Delta y},\\ c_{ij}^{10} & \;=\frac{-y{{\;}^{\prime }}_{j+1}p(x{{\;}^{\prime }}_{i},y{{\;}^{\prime }}_{j})+y{{\;}^{\prime }}_{j}p(x{{\;}^{\prime }}_{i},y{{\;}^{\prime }}_{j+1})+y{{\;}^{\prime }}_{j+1}p(x{{\;}^{\prime }}_{i+1},y{{\;}^{\prime }}_{j})-y{{\;}^{\prime }}_{j}p(x{{\;}^{\prime }}_{i+1},y{{\;}^{\prime }}_{j+1})}{\Delta x\Delta y},\\ c_{ij}^{01} & \;=\frac{-x{{\;}^{\prime }}_{i+1}p(x{{\;}^{\prime }}_{i},y{{\;}^{\prime }}_{j})+x{{\;}^{\prime }}_{i+1}p(x{{\;}^{\prime }}_{i},y{{\;}^{\prime }}_{j+1})+x{{\;}^{\prime }}_{i}p(x{{\;}^{\prime }}_{i+1},y{{\;}^{\prime }}_{j})-x{{\;}^{\prime }}_{i}p(x{{\;}^{\prime }}_{i+1},y{{\;}^{\prime }}_{j+1})}{\Delta x\Delta y}\\ \mathrm{and}\;c_{ij}^{11} & \;=\frac{p(x{{\;}^{\prime }}_{i},y{{\;}^{\prime }}_{j})-p(x{{\;}^{\prime }}_{i},y{{\;}^{\prime }}_{j+1})-p(x{{\;}^{\prime }}_{i+1},y{{\;}^{\prime }}_{j})+p(x{{\;}^{\prime }}_{i+1},y{{\;}^{\prime }}_{j+1})}{\Delta x\Delta y}. \end{array}$$

An arbitrary *p*(*x*′,*y*′) is then approximated over *R* via superposition of the interpolated loads over each subdomain:

3.5 $$p(x^{\prime },y^{\prime })\approx b(x^{\prime },y^{\prime })\equiv \sum_{i=1}^{M}\sum_{j=1}^{N}b_{ij}(x^{\prime },y^{\prime }),$$

where the bilinear interpolant over subdomain *R*_*ij*_ is defined as follows:

3.6 $$b_{ij}(x^{\prime },y^{\prime })\equiv \left\{ \begin{array}{ll} \sum_{m=0}^{1}\sum_{n=0}^{1}c_{ij}^{mn}{\left(x^{\prime }\right)}^{m}{\left(y^{\prime }\right)}^{n} & \text{for }(x^{\prime },y^{\prime })\in R_{ij},\\ 0 & \text{for }(x^{\prime },y^{\prime })\notin R_{ij}. \end{array}\right.$$

In turn, the potentials corresponding to the polynomial component (*x*′)^*m*^(*y*′)^*n*^ over *R*_*ij*_ are defined as follows:

3.7 $${\text{A}}_{ij}^{mn}=\int_{y_{j}}^{y_{j+1}}\int_{x_{i}}^{x_{i+1}}\frac{{\left(x^{\prime }\right)}^{m}{\left(y^{\prime }\right)}^{n}}{r}\;dx^{\prime }\;dy^{\prime }$$

and

3.8 $${\text{B}}_{ij}^{mn}=\int_{y_{j}}^{y_{j+1}}\int_{x_{i}}^{x_{i+1}}{\left(x^{\prime }\right)}^{m}{\left(y^{\prime }\right)}^{n}\log(z+r)\;dx^{\prime }\;dy^{\prime }.$$

Equations (3.7) and (3.8) are special cases of (3.2) and (3.3), respectively, with specified traction distributions over a given subdomain. We then have that ${\text{A}}_{ij}^{mn}=\partial {\text{B}}_{ij}^{mn}/\partial z$, and both satisfy the Laplace equation within the whole of the half-space (i.e. $\Delta {\text{A}}_{ij}^{mn}=\Delta {\text{B}}_{ij}^{mn}=0$). The potentials are then written for the interpolated loads *b*_*ij*_(*x*′,*y*′)

3.9 $$V_{ij}\equiv \sum_{m=0}^{1}\sum_{n=0}^{1}c_{ij}^{mn}{\text{A}}_{ij}^{mn}$$

and

3.10 $$\chi_{ij}\equiv \sum_{m=0}^{1}\sum_{n=0}^{1}c_{ij}^{mn}{\text{B}}_{ij}^{mn}.$$

By superposition, equations (3.2) and (3.3) are approximated over the entire domain *R* as follows:

3.11 $$V\approx \sum_{i=1}^{M}\sum_{j=1}^{N}V_{ij}$$

and

3.12 $$\chi \approx \sum_{i=1}^{M}\sum_{j=1}^{N}\chi_{ij}.$$

The derivatives of the potentials in equations (3.11) and (3.12) can be constructed from the derivatives of those in equations (3.7) and (3.8), respectively, in the same manner. Appendix B contains the complete set of closed-form solutions to equations (3.7) and (3.8) and the spatial derivatives required to satisfy the stress, strain and displacement formulae presented in appendix A.

### 3.2. Example calculation for bilinear boundary conditions

Next, let us consider a brief example of the calculation strategy that led to these expressions (in contrast with those reported in [7,11]). Consider the following function:

3.13 $$\frac{\partial^{2}{\text{B}}_{ij}^{11}}{\partial x^{2}}=\frac{\partial^{2}}{\partial x^{2}}\int_{y_{j}}^{y_{j+1}}\int_{x_{i}}^{x_{i+1}}x^{\prime }y^{\prime }\log(z+r)\;dx^{\prime }\;dy^{\prime }.$$

This function would appear in equations (A 4) and (A 10) for *x*-directional strain and stress, respectively. It is convenient to pair the spatial and integration coordinates as they appear in the distance function in equation (3.1), as substitution allows one to cancel the integrals and derivatives in the calculations with a change of sign. As (*x*′−*x*)(*y*′−*y*)=*x*′*y*′−*yx*′−*xy*′+*xy*, we see that we may write equation (3.13) as

$$\begin{array}{rl} \frac{\partial^{2}{\text{B}}_{ij}^{11}}{\partial x^{2}} & \;=\frac{\partial^{2}}{\partial x^{2}}\left(\int_{y_{j}}^{y_{j+1}}\int_{x_{i}}^{x_{i+1}}(x^{\prime }-x)(y^{\prime }-y)\log(z+r)\;dx^{\prime }\;dy^{\prime }\right)+\frac{\partial^{2}}{\partial x^{2}}\left(y\int_{y_{j}}^{y_{j+1}}\int_{x_{i}}^{x_{i+1}}x^{\prime }\log(z+r)\;dx^{\prime }\;dy^{\prime }\right)\\ & \;\;+\frac{\partial^{2}}{\partial x^{2}}\left(x\int_{y_{j}}^{y_{j+1}}\int_{x_{i}}^{x_{i+1}}y^{\prime }\log(z+r)\;dx^{\prime }\;dy^{\prime }\right)-\frac{\partial^{2}}{\partial x^{2}}\left(xy\int_{y_{j}}^{y_{j+1}}\int_{x_{i}}^{x_{i+1}}\log(z+r)\;dx^{\prime }\;dy^{\prime }\right)\\ & \;=\frac{\partial^{2}}{\partial x^{2}}\int_{y_{j}}^{y_{j+1}}\int_{x_{i}}^{x_{i+1}}(x^{\prime }-x)(y^{\prime }-y)\log(z+r)\;dx^{\prime }\;dy^{\prime }+y\frac{\partial^{2}{\text{B}}_{ij}^{10}}{\partial x^{2}}+x\frac{\partial^{2}{\text{B}}_{ij}^{01}}{\partial x^{2}}+2\frac{\partial {\text{B}}_{ij}^{01}}{\partial x}\\ & \;\;-xy\frac{\partial^{2}{\text{B}}_{ij}^{00}}{\partial x^{2}}-2y\frac{\partial {\text{B}}_{ij}^{00}}{\partial x}. \end{array}$$

The first term of the right-hand side of the above expression is then calculated as follows:

$$\begin{array}{rl} & \frac{\partial^{2}}{\partial x^{2}}\int_{y_{j}}^{y_{j+1}}\int_{x_{i}}^{x_{i+1}}(x^{\prime }-x)(y^{\prime }-y)\log(z+r)\;dx^{\prime }\;dy^{\prime }\\ & \;\;=\int_{y_{j}}^{y_{j+1}}{\left[\frac{{\left(x^{\prime }-x\right)}^{2}(y^{\prime }-y)}{r(z+r)}+(y^{\prime }-y)\log(z+r)]\right|}_{x_{i}}^{x_{i+1}}\;dy^{\prime }\\ & \;\;={{\left[\frac{1}{2}zr+\frac{1}{2}(3{\left(x^{\prime }-x\right)}^{2}+{\left(y^{\prime }-y\right)}^{2})\log(z+r)]\right|}_{x_{i}}^{x_{i+1}}|}_{y_{j}}^{y_{j+1}}. \end{array}$$

A closed-form solution of the required second partial derivative of ${\text{B}}_{ij}^{11}$ is then shown to be:

$$\begin{array}{rl} \frac{\partial^{2}{\text{B}}_{ij}^{11}}{\partial x^{2}} & \;={{\left[\frac{1}{2}zr+\frac{1}{2}(3{\left(x^{\prime }-x\right)}^{2}+{\left(y^{\prime }-y\right)}^{2})\log(z+r)]\right|}_{x_{i}}^{x_{i+1}}|}_{y_{j}}^{y_{j+1}}+y\frac{\partial^{2}{\text{B}}_{ij}^{10}}{\partial x^{2}}+x\frac{\partial^{2}{\text{B}}_{ij}^{01}}{\partial x^{2}}+\cdots \\ & \;2\frac{\partial {\text{B}}_{ij}^{01}}{\partial x}-xy\frac{\partial^{2}{\text{B}}_{ij}^{00}}{\partial x^{2}}-2y\frac{\partial {\text{B}}_{ij}^{00}}{\partial x}, \end{array}$$

where the derivatives of the potentials with respect to the lower-order pressure fields are already determined, as in appendix B. Owing to the symmetry of ${\text{B}}_{ij}^{11}$, $\partial^{2}{\text{B}}_{ij}^{11}/\partial y^{2}$ can be immediately deduced as follows:

$$\begin{array}{rl} \frac{\partial^{2}{\text{B}}_{ij}^{11}}{\partial y^{2}} & \;={{\left[\frac{1}{2}zr+\frac{1}{2}(3{\left(y^{\prime }-y\right)}^{2}+{\left(x^{\prime }-x\right)}^{2})\log(z+r)]\right|}_{x_{i}}^{x_{i+1}}|}_{y_{j}}^{y_{j+1}}+y\frac{\partial^{2}{\text{B}}_{ij}^{10}}{\partial y^{2}}+x\frac{\partial^{2}{\text{B}}_{ij}^{01}}{\partial y^{2}}+\cdots \\ & \;2\frac{\partial {\text{B}}_{ij}^{10}}{\partial y}-xy\frac{\partial^{2}{\text{B}}_{ij}^{00}}{\partial y^{2}}-2x\frac{\partial^{2}{\text{B}}_{ij}^{00}}{\partial y}. \end{array}$$

A third result can be obtained from the calculation above by considering some facts about the potentials involved. For instance, we know that $\partial^{2}{\text{B}}_{ij}^{11}/\partial z^{2}=\partial {\text{A}}_{ij}^{11}/\partial z$ by the definition for the potentials in equations (3.7) and (3.8). Owing to the fact that these are harmonic functions, we can therefore write the following:

$$\frac{\partial {\text{A}}_{ij}^{11}}{\partial z}=-\frac{\partial^{2}{\text{B}}_{ij}^{11}}{\partial x^{2}}-\frac{\partial^{2}{\text{B}}_{ij}^{11}}{\partial y^{2}}={{\left[zr]\right|}_{x_{i}}^{x_{i+1}}|}_{y_{j}}^{y_{j+1}}+x\frac{\partial {\text{A}}_{ij}^{01}}{\partial z}+y\frac{\partial {\text{A}}_{ij}^{10}}{\partial z}-xy\frac{\partial {\text{A}}_{ij}^{00}}{\partial z}.$$

This provides a closed-form solution for the expression appearing in the vertical displacement and strain, as well as all three normal stresses in appendix A. The result can be verified by direct calculation and/or relevant solutions from appendix B. All other calculations for the required closed-form solutions proceeded in analogous ways.

## 4. Convergence and error assessment

Convergence of the proposed discretization scheme is investigated for interpolation of an arbitrary pressure field and associated error. We define an error function across the discretized loaded region as the differences between an exact boundary condition and the approximation in equation (3.5):

4.1 $$e(x^{\prime },y^{\prime })=|p(x^{\prime },y^{\prime })-b(x^{\prime },y^{\prime })|.$$

We have been unable to derive an analytical bound on the error function given in equation (4.1) or find one in the literature pertaining to bilinear interpolation. Thus, we opt to directly assess errors associated with the proposed interpolation scheme in a convergence study. Consider the following biquadratic pressure distribution:

4.2 $$p(x^{\prime },y^{\prime })=\frac{9}{4}\left(1-{\left(\frac{x^{\prime }}{a}\right)}^{2}\right)\left(1-{\left(\frac{y^{\prime }}{a}\right)}^{2}\right).$$

**Figure 3.**
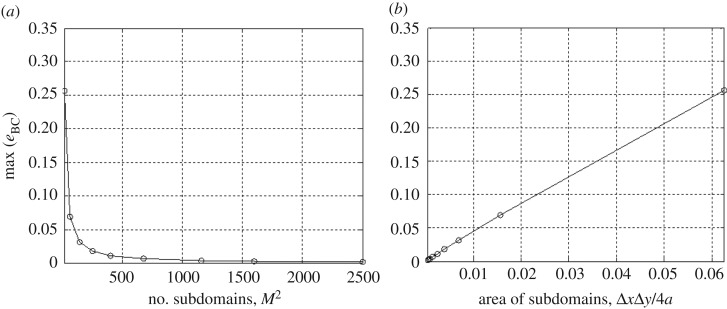
Convergence of maximum error of the bilinear interpolant to the biquadratic function in equation (4.2), with respect to (*a*) the total number of subdomains, and (*b*) the area of subdomains. Results are for discretization schemes ranging from *M*=*N*=4 to *M*=*N*=50.

This distribution is defined over a square area, *R*_*s*_={(*x*′,*y*′,0)|−*a*≤*x*′≤*a* ,−*a*≤*y*′≤*a*}. The constant coefficient $\frac{9}{4}$ is conveniently used to normalize the pressure profile by an average pressure over the square region. Let *b*(*x*′,*y*′) be the piecewise bilinear interpolant of *p*(*x*′,*y*′) over *M*^2^ subdomains, as defined by equations (3.5) and (3.6). Figure 3 shows the convergence rate of the bilinear interpolant to the boundary conditions given by equation (4.2) with respect to the total number of subdomains and their area versus the maximum error values in equation (4.1). Similarly, we find that the solution errors relative to the exact solution for the vertical surface displacements, (i.e. *e*_*w*_(*x*,*y*)=|*w*_exact_(*x*,*y*)−*w*_approx_(*x*,*y*)|) show the same convergence rate of the boundary conditions in equation (4.1).

## 5. Numerical example

Having established a procedure for determining stress and displacement in an elastic half-space under arbitrary pressure boundary conditions, we present a numerical example of its application. When external forces act on a rigid footing with a rough surface resting on a granular material, an apparent area of contact forms along the geometry of the foundation. The resulting equilibrium state, and ultimately the foundation settlement, are dependent upon the system variables (e.g. geometry and loading history [35]) in relation to contact phenomena that occur at the interfaces of the footing and supporting granular soils. To produce a mathematical description within the proposed scope of the present study, we deliberately reduce the multitude of soil–structure interaction phenomena to a single quasi-static boundary-value problem referring only to phenomenological observations at the foundation scale. We assume a stress-free reference as per the theory of elasticity; the theory applied here assumes no body forces and is therefore incapable of modelling geostatic stresses. In the following, we carefully develop a mathematical expression for vertical traction boundary conditions based on laboratory-scale experimental data available from the literature.

Recall the discussion of boundary conditions in §3. Of particular interest are Murzenko's results [21], which exhibit a dip in pressure at the centre of the loaded domain, as shown in figure 2. In the following, we empirically describe a two-dimensional vertical traction distribution that exactly fits these point-value measurements over a square contact area. We also impose zero values of normal traction along the edge of the square region. This enforcement both satisfies zero shear resistances along the edges of the surface footing and maintains continuity in the traction conditions across the entirety of the boundary plane. Otherwise, the discontinuities across the boundary of the loaded region lead to singularities in the resulting stress field [7].

### 5.1. The generation of an empirical pressure surface

Considering a square loaded region *R*_*s*_ of half-width *a*, we begin to empirically prescribe traction boundary conditions with a parametrized function of a single variable *ρ*:

5.1 $$p(\rho )=A\cos{\left(\frac{\pi }{2}\frac{\rho }{a}\right)}^{\omega }-B\;\exp\left(-{\left(\frac{\rho }{\sigma a}\right)}^{2}\right).$$

This function was originally developed by Ai *et al*. [38] to interpolate stress data measured in sandpiles. As already stated, there is a marked resemblance between Murzenko's data and these sandpile stresses with respect to shape and variation. Based on the results of this review, it was determined that a simple curve fitting of Murzenko's data by equation (5.1) provides a rudimentary approximation of the boundary conditions. The values of the free parameters *A*, *B*, *ω* and *σ* are calculated to fit equation (5.1) for empirical data regarding contact pressure distribution. The four data points (including the zero edge values) across the centre line or diagonal of a square domain are paired with these unknown parameters, which can be solved with a nonlinear equation solver (e.g. the Matlab ‘fsolve’ function).

**Figure 4.**
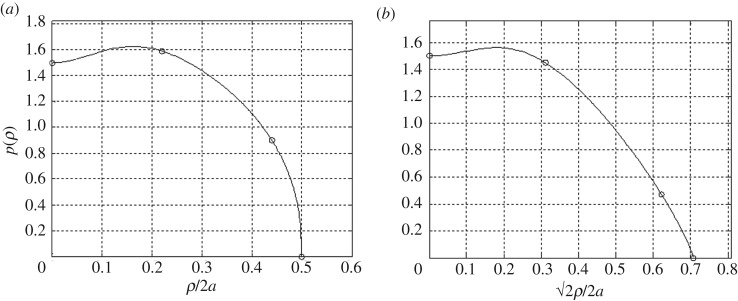
Curve-fit representations of equation (5.1) with respect to Murzenko's experimental data [21] for the case where he reported an average pressure of approximately 1 kgf cm^−2^, (*a*) across the centre line, and (*b*) across the diagonal line of the square footing.

With reference to the curve fitting across the 0° (centre) and 45° (diagonal) angles of the domain, we define two sets of parameters, i.e. *A*_0_, *B*_0_, *ω*_0_, *σ*_0_, *A*_45_, *B*_45_, *ω*_45_ and *σ*_45_. We select the case which Murzenko reported as having an average pressure 1 kgf cm^−2^, depicted in figure 2, for curve-fitting using equation (5.1). We report that there are a number of combinations of parameter values for equation (5.1) which fit the values with slight variations in the shape of the curve; in other words, the curve-fit is not unique. The selected distributions are shown in figure 4, and the relevant parameters are listed in table 1. Although this parametric fitting provides us some understanding of the contact phenomena, it is unknown exactly how the contact stresses vary between these two lines. In an attempt to continuously describe variations between the two lines in a Cartesian domain over a square area, we use coordinate transformation from a mapping function:

5.2 $$f(\theta )=\min\left[\frac{1}{|\cos\theta |},\frac{1}{|\sin\theta |}\right].$$

This function varies from 1 to $\sqrt{2}$ such that *f*(*θ*) is the distance from the centre of a unit square to its boundary at angle *θ*. Taking the variable *ρ* to be radial, and letting *α*(*θ*)=*a*⋅*f*(*θ*), we map the line load to a square domain by letting *a*→*α*(*θ*) in equation (5.1). In turn, functions of *θ* can be defined for all the curve-fitting parameters given in equation (5.1) as follows:

$$\begin{array}{rl} A(\theta ) & \;=A_{0}f{\left(\theta \right)}^{2\log(A_{45}/A_{0})/\log(2)},\\ B(\theta ) & \;=B_{0}f{\left(\theta \right)}^{2\log(B_{45}/B_{0})/\log(2)},\\ \omega (\theta ) & \;=\omega_{0}f{\left(\theta \right)}^{2\log(\omega_{45}/\omega_{0})/\log(2)}\\ \mathrm{and}\;\sigma (\theta ) & \;=\sigma_{0}f{\left(\theta \right)}^{2\log(\sigma_{45}/\sigma_{0})/\log(2).} \end{array}$$

**Table 1.** Input parameters for equation (5.3) fitting the Murzenko data in the case with reported average pressure 1 kgf cm^−2^.

*A*_0_	*A*_45_	*B*_0_	*B*_45_	*ω*_0_	*ω*_45_	*σ*_0_	*σ*_45_
1.7939	1.8292	0.2939	0.3292	0.4119	0.8114	0.2715	0.2853

These functions vary continuously from a given curve-fit parameter at 0° to that at 45°. Transforming these auxiliary functions to a Cartesian coordinate system (i.e. letting $\theta =\tan^{-1}(y^{\prime }/x^{\prime })$, $\rho =\sqrt{x{{\;}^{\prime }}^{2}+y{{\;}^{\prime }}^{2}}$), we can write the vertical traction as follows:

5.3 $$p(x^{\prime },y^{\prime })=A(x^{\prime },y^{\prime })\cos{\left(\frac{\pi }{2}\frac{\sqrt{x{{\;}^{\prime }}^{2}+y{{\;}^{\prime }}^{2}}}{\alpha (x^{\prime },y^{\prime })}\right)}^{\omega (x^{\prime },y^{\prime })}-B(x^{\prime },y^{\prime })\exp\left(-{\left(\frac{\sqrt{x{{\;}^{\prime }}^{2}+y{{\;}^{\prime }}^{2}}}{\sigma (x^{\prime },y^{\prime })\alpha (x^{\prime },y^{\prime })}\right)}^{2}\right).$$

**Figure 5.**
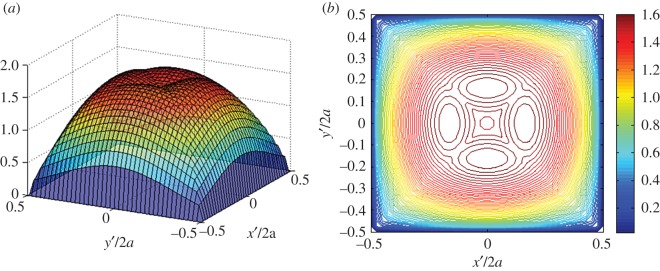
(*a*) A surface plot of a pressure distribution with pronounced pressure dip, derived to fit the data from Murzenko [21] (for the case with reported average pressure 1 kgf cm^−2^) by way of equation (5.3). (*b*) A contour plot of the same (note that the average pressure under this surface is calculated to be ≈1.095 kgf cm^−2^).

The results of equation (5.3) are shown in figure 5. The function exactly predicts all five experimental data points (along with zero values at the boundary) with the parameters in table 1, and reveals a continuous traction field across the entire loaded domain. However, there is a discrepancy in comparison to the total force reported in the literature:

$$\frac{|P-P_{M}|}{P}\approx 8.6\%,$$

where $P=\int_{-a}^{a}\int_{-a}^{a}p(x^{\prime },y^{\prime })\;dx^{\prime }\;dy^{\prime }$ is the total force of the continuous surface traction of equation (5.3), and *P*_*M*_ denotes the applied load as per the average pressure, 1 kgf cm^−2^, reported in [21]. There are a number of possible reasons for this discrepancy, one being the extrapolation of the boundary traction between the centre and diagonal lines. However, Murzenko recognized similar discrepancies in total force between his curve-fitted surface tractions and the measurements of applied load, although his method of obtaining a continuous distribution of contact pressures is unknown.

### 5.2. Calculations for displacement, strain and stress fields

We note that the closed form solutions in appendix B may appear to be undefined at the boundary *z*=0. However, given the boundary conditions presented here, these expressions all tend to finite limits on the boundary. The values of the limits at these points are applied so that the displacement, stress and strain fields are all continuous at all points within the body and upon its boundary. Specific to the traction boundary condition described by equation (5.3), the vertical surface displacements are solved using equation (A 3):

$$w(x,y,0)=\frac{\lambda +2\mu }{\mu (\lambda +\mu )}\frac{V(x,y,0)}{4\pi }=\frac{\left(1-\nu^{2}\right)}{E\pi }V(x,y,0),$$

where *E* and *ν* are the Young's modulus and Poisson's ratio of the elastic material, respectively. The potential *V* is given by equation (3.2). After normalization with respect to the elastic constants and width (2*a*) of the square footing, we have the following:

5.4 $$w^{*}(x^{\prime },y^{\prime })\equiv \frac{E}{2a(1-\nu^{2})}w(x^{\prime },y^{\prime },0)=\frac{V(x^{\prime },y^{\prime },0)}{2a\pi }.$$

**Figure 6.**
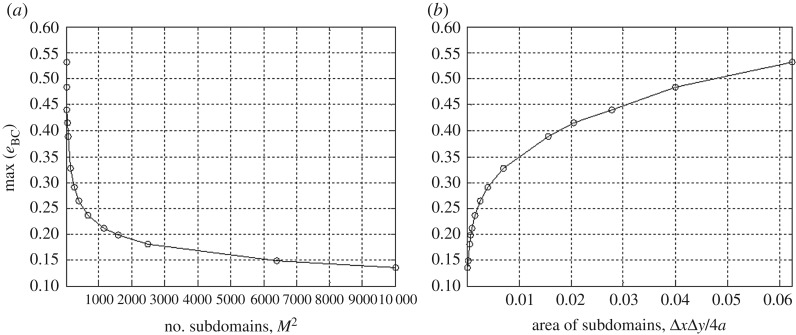
Convergence of the bilinear interpolant to the empirical boundary condition shown in figure 5, with respect to (*a*) the total number of subdomains and (*b*) the area of subdomains. Results are for discretization schemes ranging from *M*=*N*=4 to *M*=*N*=100.

**Figure 7.**
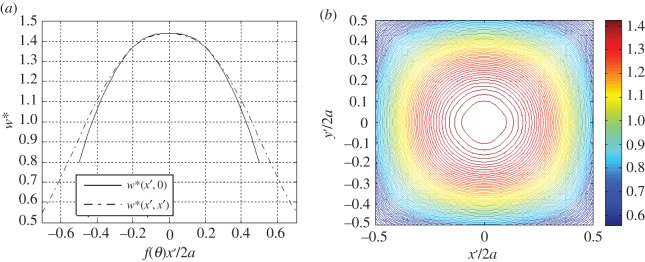
(*a*) Cross-sectional and (*b*) contour plots of the normalized displacement field of equation (5.4) specific to the vertical traction boundary condition of figure 5.

The normalized displacement field can be calculated by the sum of the potential functions of equation (3.9) over 40×40 discretized subdomains (i.e. $w^{*}(x^{\prime },y^{\prime })\approx (1/2\pi a)\sum_{i=1}^{40}\sum_{j=1}^{40}V_{ij}$). The loaded domain is discretized with this number of subdomains by trial and error to satisfy the convergence criterion presented in figure 6. We note that the higher-order boundary condition applied here shows a slower rate of convergence than the biquadratic function in §4. A normalized displacement field over *R*_*s*_ of equation (5.4) is shown in figure 7.

Recalling the discussion in §2, a symmetrically loaded rigid plate resting on a homogeneous material will experience uniform surface displacement, while the Neumann problem outlined in equation (2.4) will generally yield non-uniform surface displacements for a given traction distribution. Considering the compatibility requirement, we interpret the results of equation (5.4) as the distribution of non-dimensionalized resistance:

5.5 $$k(x^{\prime },y^{\prime })=\frac{p(x^{\prime },y^{\prime })}{w^{*}(x^{\prime },y^{\prime })}.$$

**Figure 8.**
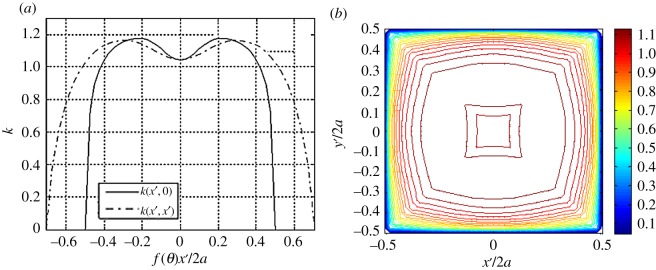
(*a*) Cross-sectional and (*b*) contour plots of the resistance field of equation (5.5) specific to the vertical traction boundary condition of figure 5.

The results from equation (5.5) are shown in figure 8. This expression of soil resistance can be viewed as a continuous distribution of elastic spring stiffnesses that is analogous to an extended Winkler foundation model.

**Figure 9.**
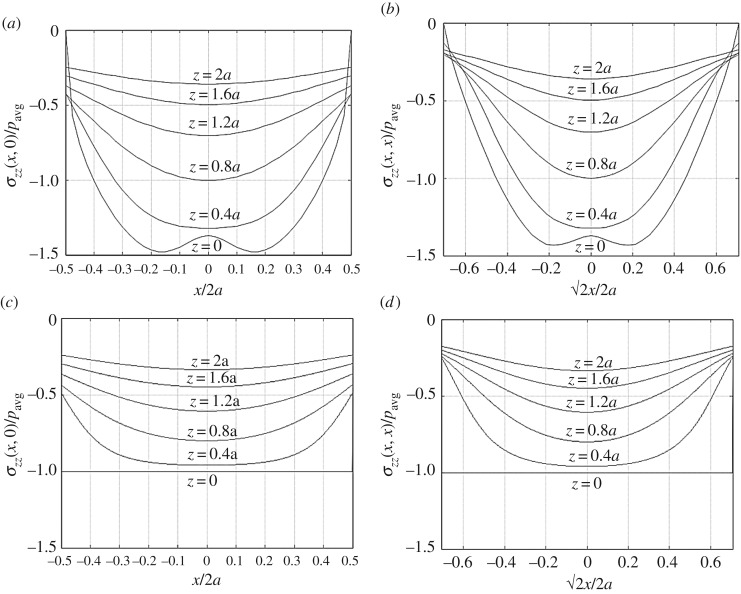
Variation of vertical stress at different depths under the semi-empirical boundary conditions (*a*) across the centre, and (*b*) across the diagonal of the loaded area. Compare with the results for a uniform average pressure on the boundary, for (*c*) the centre and (*d*) the diagonal. The results are normalized by the average pressure.

**Figure 10.**
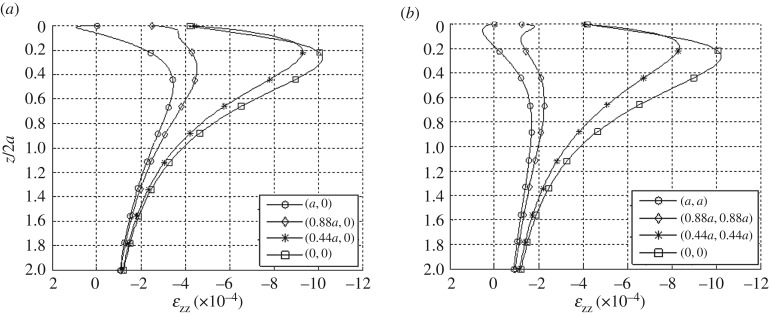
Vertical strains with depth under points across (*a*) the centre line, and (*b*) the diagonal of the loaded region. We choose example elastic constants *E*=1000 kgf cm^−2^, *ν*=0.4 for this calculation.

The vertical stresses in the elastic body are independent of the elastic constants from equation (A 12). Notably, *σ*_*zz*_ at *z*=0 is exactly equal to, but opposite in sign of, the described vertical tractions. Figure 9 compares vertical stresses along depths beneath the loaded area with the classical solution obtained from a uniform pressure boundary condition [6,7]. The non-uniform pressure boundary condition yields higher stresses near the centre and lower stresses towards the edge of the loaded area. In addition, the vertical strains produced within the body from the applied tractions can be obtained from equation (A 6). For illustration purposes only, vertical strain distributions along depth are plotted at the locations of Murzenko's pressure measurements in figure 10. The values of Young's modulus and Poisson's ratio are arbitrarily selected (e.g. values of tri-axial compression tests on very dense sand found in soil mechanics textbooks, [43,44]). Similar to strain-influence methods [9], the definite integration of each curve from the surface to an infinite depth yields displacement from equation (A 3) at Murzenko's sampling locations. The applied surface tractions yield an interesting trend in vertical strain fields; tensile strains are pronounced beneath the corners mainly due to the Poisson effect and the zero traction (resistance) boundary condition imposed on the edges of the loaded domain.

The results for displacement, stress and strain, and in particular the resulting non-uniform distribution of resistance/spring stiffness, correspond uniquely to the traction boundary condition, which is largely dependent upon the nature of the granular soil. The proposed solution could be further refined to incorporate mobilized shear resistance of the particles, such as the discrete models presented by Pasternak [45] and Kerr [30]. In particular, geostatic stress states may introduce another set of initial and/or boundary conditions to foundation systems.

It is important to note that this particular distribution of contact forces *p*(*x*′,*y*′) should not be considered conclusively supportive of a particular model of stress propagation in granular materials, either hyperbolic or elliptic [46]. It is also true that describing the supporting soil medium as an isotropic, homogeneous elastic material with a stress-free reference state is unlikely to produce contact stress distributions like those measured in Murzenko's experiment and/or the sandpile tests. In fact, there is no comprehensive theory that can predict contact force distribution. The stress distribution is probably the phenomenological outcome of numerous multiscale parameters. The presence and degree of inter-granular friction bonding is dependent upon grain-scale geometry, relative density state, dilation and past loading history, which in turn determine the bulk constitutive phenomena of the granular media. For these reasons it was intended only to phenomenologically prescribe a Neumann boundary condition to exemplify the proposed solution procedures. The theoretical question of whether these physical factors may be all together incorporated in the calculations remains unanswered. With more empirical data at various interrelated scales, more detailed analytical models must be developed to describe the system-scale behaviour. The characteristics of granular materials can be further contextualized by many granular physics studies [47–49].

## 6. Concluding remarks

This study has aimed to provide a general solution approach to the classical Boussinesq–Love problem for arbitrary loads over a rectangular surface of an elastic half-space, described as Neumann boundary conditions. It is very challenging to adequately describe contact phenomena in foundation systems because contact stress distributions within the loaded area of even purely elastic bodies is neither uniform nor linear [5,24,25]. The solution procedure presented here, along with the closed-form solutions of the potential functions, are readily applied to arbitrary traction boundary conditions in a Cartesian coordinate system. We attempt to apply these potentials to foundation engineering situations, specifically to elastic settlement analysis of shallow foundations. However, we hope that the ubiquitous nature of the Laplace equation allows the present solution to be feasibly applied to other fields of study. Further, the proposed method of solution can be applied to tangential traction boundary-value problems [50–52]. A set of bilinear solutions to this problem for rectangular regions is currently under development by the present authors.

## Supplementary Material

Source codes and data files

## Data accessibility

All data and source code required to generate the results presented here have been included as the electronic supplementary material.

## Appendix A. Formula for the displacement vector, stress and strain tensors

The displacements (*u*,*v*,*w*) in the (*x*,*y*,*z*) directions are given by

A 1 $$\begin{array}{rl} u & \;=-\frac{1}{4\pi }\left(\frac{1}{\lambda +\mu }\frac{\partial \chi }{\partial x}+\frac{z}{\mu }\frac{\partial V}{\partial x}\right), \end{array}$$

A 2 $$\begin{array}{rl} v & \;=-\frac{1}{4\pi }\left(\frac{1}{\lambda +\mu }\frac{\partial \chi }{\partial y}+\frac{z}{\mu }\frac{\partial V}{\partial y}\right) \end{array}$$

A 3 $$\begin{array}{rl} \text{and}\;w & \;=\frac{1}{4\pi \mu }\left(\frac{\lambda +2\mu }{\lambda +\mu }V-z\frac{\partial V}{\partial z}\right). \end{array}$$

Here, *λ* and *μ* are the Lame's constants. The strains are derived simply by differentiation as follows:

A 4 $$\begin{array}{rl} \varepsilon_{xx} & \;=\frac{\partial u}{\partial x}=-\frac{1}{4\pi }\left(\frac{1}{\lambda +\mu }\frac{\partial^{2}\chi }{\partial x^{2}}+\frac{z}{\mu }\frac{\partial^{2}V}{\partial x^{2}}\right), \end{array}$$

A 5 $$\begin{array}{rl} \varepsilon_{yy} & \;=\frac{\partial v}{\partial y}=-\frac{1}{4\pi }\left(\frac{1}{\lambda +\mu }\frac{\partial^{2}\chi }{\partial y^{2}}+\frac{z}{\mu }\frac{\partial^{2}V}{\partial y^{2}}\right), \end{array}$$

A 6 $$\begin{array}{rl} \varepsilon_{zz} & \;=\frac{\partial w}{\partial z}=\frac{1}{4\pi }\left(\frac{1}{\lambda +\mu }\frac{\partial V}{\partial z}-\frac{z}{\mu }\frac{\partial^{2}V}{\partial z^{2}}\right), \end{array}$$

A 7 $$\begin{array}{rl} \varepsilon_{xz} & \;=\frac{1}{2}\left(\frac{\partial u}{\partial z}+\frac{\partial w}{\partial x}\right)=\frac{-1}{4\pi \mu }\left(z\frac{\partial^{2}V}{\partial x\partial z}\right), \end{array}$$

A 8 $$\begin{array}{rl} \varepsilon_{yz} & \;=\frac{1}{2}\left(\frac{\partial v}{\partial z}+\frac{\partial w}{\partial x}\right)=\frac{-1}{4\pi \mu }\left(z\frac{\partial^{2}V}{\partial y\partial z}\right) \end{array}$$

A 9 $$\begin{array}{rl} \text{and}\;\varepsilon_{xy} & \;=\frac{1}{2}\left(\frac{\partial u}{\partial y}+\frac{\partial v}{\partial x}\right)=\frac{-1}{4\pi }\left(\frac{1}{\lambda +\mu }\frac{\partial^{2}\chi }{\partial x\partial y}+\frac{z}{\mu }\frac{\partial^{2}V}{\partial x\partial y}\right). \end{array}$$

The stresses are then derived from the generalized three-dimensional Hooke's Law, expressed in simplified form as

A 10 $$\begin{array}{rl} \sigma_{xx} & \;=\frac{1}{2\pi }\left(\frac{\lambda }{\lambda +\mu }\frac{\partial V}{\partial z}-\frac{\mu }{\lambda +\mu }\frac{\partial^{2}\chi }{\partial x^{2}}-z\frac{\partial^{2}V}{\partial x^{2}}\right), \end{array}$$

A 11 $$\begin{array}{rl} \sigma_{yy} & \;=\frac{1}{2\pi }\left(\frac{\lambda }{\lambda +\mu }\frac{\partial V}{\partial z}-\frac{\mu }{\lambda +\mu }\frac{\partial^{2}\chi }{\partial y^{2}}-z\frac{\partial^{2}V}{\partial y^{2}}\right), \end{array}$$

A 12 $$\begin{array}{rl} \sigma_{zz} & \;=\frac{1}{2\pi }\left(\frac{\partial V}{\partial z}-z\frac{\partial^{2}V}{\partial z^{2}}\right), \end{array}$$

A 13 $$\begin{array}{rl} \sigma_{xz} & \;=\frac{-1}{2\pi }\left(z\frac{\partial^{2}V}{\partial x\partial z}\right), \end{array}$$

A 14 $$\begin{array}{rl} \sigma_{yz} & \;=\frac{-1}{2\pi }\left(z\frac{\partial^{2}V}{\partial y\partial z}\right) \end{array}$$

A 15 $$\begin{array}{rl} \text{and}\;\sigma_{xy} & \;=\frac{-1}{2\pi }\left(\frac{\mu }{\lambda +\mu }\frac{\partial^{2}\chi }{\partial x\partial y}+z\frac{\partial^{2}V}{\partial x\partial y}\right). \end{array}$$

## Appendix B. Closed-form solutions of potential functions

We here list the closed-form solutions of the potentials for arbitrary rectangular region *R* under the simple polynomial loads employed throughout this work. We define

$$\begin{array}{rl} {\text{A}}^{mn} & \;\equiv \iint_{R}\frac{{\left(x^{\prime }\right)}^{m}{\left(y^{\prime }\right)}^{n}}{r}\;dx^{\prime }\;dy^{\prime }\\ \mathrm{and}\;{\text{B}}^{mn} & \;\equiv \iint_{R}{\left(x^{\prime }\right)}^{m}{\left(y^{\prime }\right)}^{n}\log(z+r)\;dx^{\prime }\;dy^{\prime }. \end{array}$$

Note that, in the following, the notation $[\;]{|}_{a_{2}}^{a_{1}}{|}_{b_{2}}^{b_{1}}$ is used to compact the multiple repeated terms generated by the double definite integration by the fundamental theorem of calculus. In general, if *F*(*x*′,*y*′) is a function of two variables, the operator acts upon it as follows:

$$[F(x^{\prime },y^{\prime })]{|}_{a_{2}}^{a_{1}}{|}_{b_{2}}^{b_{1}}=(F(a_{1},b_{1})-F(a_{1},b_{2}))-(F(a_{2},b_{1})-F(a_{2},b_{2})).$$

**B.1. Constant load solutions**

$$\begin{array}{rl} {\text{A}}^{00} & \;={{\left[(y^{\prime }-y)\log(r+x^{\prime }-x)+(x^{\prime }-x)\log(r-y+y^{\prime })-z\tan^{-1}\left(\frac{\left(x^{\prime }-x)(y^{\prime }-y\right)}{zr}\right)]\right|}_{a_{2}}^{a_{1}}|}_{b_{2}}^{b_{1}}\\ \frac{\partial {\text{A}}^{00}}{\partial x} & \;={{\left[-\log(r+y^{\prime }-y)]\right|}_{a_{2}}^{a_{1}}|}_{b_{2}}^{b_{1}}\\ \frac{\partial^{2}{\text{A}}^{00}}{\partial x^{2}} & \;={{\left[-\frac{\left(x^{\prime }-x)(y^{\prime }-y\right)}{({\left(x^{\prime }-x\right)}^{2}+z^{2})r}]\right|}_{a_{2}}^{a_{1}}|}_{b_{2}}^{b_{1}}\\ \frac{\partial {\text{A}}^{00}}{\partial y} & \;={{\left[-\log((x^{\prime }-x)+r)]\right|}_{a_{2}}^{a_{1}}|}_{b_{2}}^{b_{1}}\\ \frac{\partial^{2}{\text{A}}^{00}}{\partial y^{2}} & \;={{\left[-\frac{\left(x^{\prime }-x)(y^{\prime }-y\right)}{({\left(y^{\prime }-y\right)}^{2}+z^{2})r}]\right|}_{a_{2}}^{a_{1}}|}_{b_{2}}^{b_{1}}\\ \frac{\partial {\text{A}}^{00}}{\partial z} & \;={{\left[-\tan^{-1}\left(\frac{\left(x^{\prime }-x)(y^{\prime }-y\right)}{zr}\right)]\right|}_{a_{2}}^{a_{1}}|}_{b_{2}}^{b_{1}}\\ \frac{\partial^{2}{\text{A}}^{00}}{\partial z^{2}} & \;={{\left[\frac{\left(x^{\prime }-x)(y^{\prime }-y)(r^{2}+z^{2}\right)}{({\left(x^{\prime }-x\right)}^{2}+z^{2})({\left(y^{\prime }-y\right)}^{2}+z^{2})r}]\right|}_{a_{2}}^{a_{1}}|}_{b_{2}}^{b_{1}}\\ \frac{\partial^{2}{\text{A}}^{00}}{\partial x\partial z} & \;={{\left[\frac{(y^{\prime }-y)z}{({\left(x^{\prime }-x\right)}^{2}+z^{2})r}]\right|}_{a_{2}}^{a_{1}}|}_{b_{2}}^{b_{1}}\\ \frac{\partial^{2}{\text{A}}^{00}}{\partial y\partial z} & \;={{\left[\frac{(x^{\prime }-x)z}{({\left(y^{\prime }-y\right)}^{2}+z^{2})r}]\right|}_{a_{2}}^{a_{1}}|}_{b_{2}}^{b_{1}}\\ \frac{\partial^{2}{\text{A}}^{00}}{\partial x\partial y} & \;={{\left[\frac{1}{r}]\right|}_{a_{2}}^{a_{1}}|}_{b_{2}}^{b_{1}}\\ {\text{B}}^{00} & \;={{\left[\begin{array}{l} -\frac{3}{2}(x^{\prime }-x)(y^{\prime }-y)+\frac{1}{2}{\left(x^{\prime }-x\right)}^{2}\left(\tan^{-1}\left(\frac{y^{\prime }-y}{x^{\prime }-x}\right)-\tan^{-1}\left(\frac{z(y^{\prime }-y)}{(x^{\prime }-x)r}\right)\right)+\cdots \\ \;\frac{1}{2}{\left(y^{\prime }-y\right)}^{2}\left(\tan^{-1}\left(\frac{x^{\prime }-x}{y^{\prime }-y}\right)-\tan^{-1}\left(\frac{z(x^{\prime }-x)}{(y^{\prime }-y)r}\right)\right)-\cdots \\ \;\frac{1}{2}z^{2}\tan^{-1}\left(\frac{\left(x^{\prime }-x)(y^{\prime }-y\right)}{zr}\right)+\cdots \\ \;z(x^{\prime }-x)\log(y^{\prime }-y+r)+z(y^{\prime }-y)\log(x^{\prime }-x+r)+\cdots \\ \;(x^{\prime }-x)(y^{\prime }-y)\log(z+r) \end{array}]\right|}_{a_{2}}^{a_{1}}|}_{b_{2}}^{b_{1}}\\ \frac{\partial {\text{B}}^{00}}{\partial x} & \;={{\left[\begin{array}{l} (x^{\prime }-x)\left(\tan^{-1}\left(\frac{(y^{\prime }-y)z}{(x^{\prime }-x)r}\right)-\tan^{-1}\left(\frac{y^{\prime }-y}{x^{\prime }-x}\right)\right)-\cdots \\ \;z\log\left((y^{\prime }-y)+r\right)-(y^{\prime }-y)\log\left(z+r\right) \end{array}]\right|}_{a_{2}}^{a_{1}}|}_{b_{2}}^{b_{1}}\\ \frac{\partial^{2}{\text{B}}^{00}}{\partial x^{2}} & \;={{\left[\tan^{-1}\left(\frac{y^{\prime }-y}{x^{\prime }-x}\right)-\tan^{-1}\left(\frac{z(y^{\prime }-y)}{(x^{\prime }-x)r}\right)]\right|}_{a_{2}}^{a_{1}}|}_{b_{2}}^{b_{1}}\\ \frac{\partial {\text{B}}^{00}}{\partial y} & \;={{\left[\begin{array}{l} (y^{\prime }-y)\left(\tan^{-1}\left(\frac{(x^{\prime }-x)z}{(y^{\prime }-y)r}\right)-\tan^{-1}\left(\frac{x^{\prime }-x}{y^{\prime }-y}\right)\right)-\cdots \\ \;z\log((x^{\prime }-x)+r)-(x^{\prime }-x)\log(z+r) \end{array}]\right|}_{b_{2}}^{b_{1}}|}_{a_{2}}^{a_{1}}\\ \frac{\partial^{2}{\text{B}}^{00}}{\partial y^{2}} & \;={{\left[\tan^{-1}\left(\frac{x^{\prime }-x}{y^{\prime }-y}\right)-\tan^{-1}\left(\frac{z(x^{\prime }-x)}{(y^{\prime }-y)r}\right)]\right|}_{a_{2}}^{a_{1}}|}_{b_{2}}^{b_{1}}\\ \frac{\partial^{2}{\text{B}}^{00}}{\partial x\partial y} & \;={{\left[\log(z+r)]\right|}_{a_{2}}^{a_{1}}|}_{b_{2}}^{b_{1}} \end{array}$$

**B.2. Linear load solutions**

$$\begin{array}{rl} {\text{A}}^{10} & \;={{\left[\frac{1}{2}(y^{\prime }-y)r+\frac{1}{2}({\left(x^{\prime }-x\right)}^{2}+z^{2})\log(r+y^{\prime }-y)]\right|}_{a_{2}}^{a_{1}}|}_{b_{2}}^{b_{1}}+x{\text{A}}^{00}\\ \frac{\partial {\text{A}}^{10}}{\partial x} & \;={{\left[-(x^{\prime }-x)\log(y^{\prime }-y+r)]\right|}_{a_{2}}^{a_{1}}|}_{b_{2}}^{b_{1}}+x\frac{\partial {\text{A}}^{00}}{\partial x}+{\text{A}}^{00}\\ \frac{\partial^{2}{\text{A}}^{10}}{\partial x^{2}} & \;={{\left[\log(r+y^{\prime }-y)-\frac{{\left(x^{\prime }-x\right)}^{2}(y^{\prime }-y)}{({\left(x^{\prime }-x\right)}^{2}+z^{2})r}]\right|}_{a_{2}}^{a_{1}}|}_{b_{2}}^{b_{1}}+x\frac{\partial^{2}{\text{A}}^{00}}{\partial x^{2}}+2\frac{\partial {\text{A}}^{00}}{\partial x}\\ \frac{\partial {\text{A}}^{10}}{\partial y} & \;={{\left[-r]\right|}_{a_{2}}^{a_{1}}|}_{b_{2}}^{b_{1}}+x\frac{\partial {\text{A}}^{00}}{\partial y}\\ \frac{\partial^{2}{\text{A}}^{10}}{\partial y^{2}} & \;={{\left[\frac{\left(y^{\prime }-y\right)}{r}]\right|}_{a_{2}}^{a_{1}}|}_{b_{2}}^{b_{1}}+x\frac{\partial^{2}{\text{A}}^{00}}{\partial y^{2}}\\ \frac{\partial {\text{A}}^{10}}{\partial z} & \;={{\left[z\log(r+y^{\prime }-y)]\right|}_{a_{2}}^{a_{1}}|}_{b_{2}}^{b_{1}}+x\frac{\partial {\text{A}}^{00}}{\partial z}\\ \frac{\partial^{2}{\text{A}}^{10}}{\partial z^{2}} & \;={{\left[\frac{z^{2}}{r(r+y^{\prime }-y)}+\log(r+y^{\prime }-y)]\right|}_{a_{2}}^{a_{1}}|}_{b_{2}}^{b_{1}}+x\frac{\partial^{2}{\text{A}}^{00}}{\partial z^{2}}\\ \frac{\partial^{2}{\text{A}}^{10}}{\partial x\partial z} & \;={{\left[\frac{(x^{\prime }-x)(y^{\prime }-y)z}{({\left(x^{\prime }-x\right)}^{2}+z^{2})r}]\right|}_{a_{2}}^{a_{1}}|}_{b_{2}}^{b_{1}}+x\frac{\partial^{2}{\text{A}}^{00}}{\partial x\partial z}+\frac{\partial {\text{A}}^{00}}{\partial z}\\ \frac{\partial^{2}{\text{A}}^{10}}{\partial y\partial z} & \;={{\left[-\frac{z}{r}]\right|}_{a_{2}}^{a_{1}}|}_{b_{2}}^{b_{1}}+y\frac{\partial^{2}{\text{A}}^{00}}{\partial y\partial z}+\frac{\partial {\text{A}}^{00}}{\partial z}\\ \frac{\partial^{2}{\text{A}}^{10}}{\partial x\partial y} & \;={{\left[\frac{\left(x^{\prime }-x\right)}{r}]\right|}_{a_{2}}^{a_{1}}|}_{b_{2}}^{b_{1}}+x\frac{\partial^{2}{\text{A}}^{00}}{\partial x\partial y}+\frac{\partial {\text{A}}^{00}}{\partial y}\\ {\text{B}}^{10} & \;=\frac{1}{18}{{\left[\begin{array}{l} 6{\left(x^{\prime }-x\right)}^{3}\left(\tan^{-1}\left(\frac{\left(y^{\prime }-y\right)}{\left(x^{\prime }-x\right)}\right)-\tan^{-1}\left(\frac{(y^{\prime }-y)z}{(x^{\prime }-x)r}\right)\right)+\cdots \\ \;3z(3{\left(x^{\prime }-x\right)}^{2}+z^{2})\log(y^{\prime }-y+r)+9(y^{\prime }-y){\left(x^{\prime }-x\right)}^{2}\log(z+r)+\cdots \\ \;(y^{\prime }-y)(-{\left(y^{\prime }-y\right)}^{2}-6{\left(x^{\prime }-x\right)}^{2}+6zr+3{\left(y^{\prime }-y\right)}^{2}\log(z+r)) \end{array}]\right|}_{a_{2}}^{a_{1}}|}_{b_{2}}^{b_{1}}+x{\text{B}}^{00}\\ \frac{\partial {\text{B}}^{10}}{\partial x} & \;={{\left[\begin{array}{l} {\left(x^{\prime }-x\right)}^{2}\left(\tan^{-1}\left(\frac{(y^{\prime }-y)z}{(x^{\prime }-x)r}\right)-\tan^{-1}\left(\frac{y^{\prime }-y}{x^{\prime }-x}\right)\right)-\cdots \\ \;z(x^{\prime }-x)\log((y^{\prime }-y)+r)-(x^{\prime }-x)(y^{\prime }-y)\log(z+r)+(x^{\prime }-x)y^{\prime } \end{array}]\right|}_{a_{2}}^{a_{1}}|}_{b_{2}}^{b_{1}}+x\frac{\partial {\text{B}}^{00}}{\partial x}+{\text{B}}^{00}\\ \frac{\partial^{2}{\text{B}}^{10}}{\partial x^{2}} & \;={{\left[\begin{array}{l} 2(x^{\prime }-x)\left(\tan^{-1}\left(\frac{y^{\prime }-y}{x^{\prime }-x}\right)-\tan^{-1}\left(\frac{(y^{\prime }-y)z}{(x^{\prime }-x)r}\right)\right)+\cdots \\ \;z\log((y^{\prime }-y)+r)+(y^{\prime }-y)\log(z+r) \end{array}]\right|}_{a_{2}}^{a_{1}}|}_{b_{2}}^{b_{1}}+\;x\frac{\partial^{2}{\text{B}}^{00}}{\partial x^{2}}+2\frac{\partial {\text{B}}^{00}}{\partial x}\\ \frac{\partial {\text{B}}^{10}}{\partial y} & \;={{\left[-\frac{1}{2}(zr+({\left(x^{\prime }-x\right)}^{2}+{\left(y^{\prime }-y\right)}^{2})\log(z+r))]\right|}_{a_{2}}^{a_{1}}|}_{b_{2}}^{b_{1}}+x\frac{\partial {\text{B}}^{00}}{\partial y}\\ \frac{\partial^{2}{\text{B}}^{10}}{\partial y^{2}} & \;={{\left[(y^{\prime }-y)\log(z+r)]\right|}_{a_{2}}^{a_{1}}|}_{b_{2}}^{b_{1}}+x\frac{\partial^{2}{\text{B}}^{00}}{\partial y^{2}}\\ \frac{\partial^{2}{\text{B}}^{10}}{\partial x\partial y} & \;={{\left[(x^{\prime }-x)\log(z+r)]\right|}_{a_{2}}^{a_{1}}|}_{b_{2}}^{b_{1}}+x\frac{\partial^{2}{\text{B}}^{00}}{\partial x\partial y}+\frac{\partial {\text{B}}^{00}}{\partial y} \end{array}$$

The solutions for the potentials A^01^, B^01^ for a linear load in the *y*′ direction can be obtained directly from these solutions by permutation of the terms related to the *x* and *y* variables in the above expressions.

**B.3. Bilinear load solutions**

$$\begin{array}{rl} {\text{A}}^{11} & \;={{\left[\frac{1}{3}r^{3}]\right|}_{a_{2}}^{a_{1}}\;|}_{b_{2}}^{b_{1}}+x{\text{A}}^{01}+y{\text{A}}^{10}-xy{\text{A}}^{00}\\ \frac{\partial {\text{A}}^{11}}{\partial x} & \;={{\left[-(x^{\prime }-x)r]\right|}_{a_{2}}^{a_{1}}|}_{b_{2}}^{b_{1}}+y\frac{\partial {\text{A}}^{10}}{\partial x}+x\frac{\partial {\text{A}}^{01}}{\partial x}+{\text{A}}^{01}-xy\frac{\partial {\text{A}}^{00}}{\partial x}-y{\text{A}}^{00}\\ \frac{\partial^{2}{\text{A}}^{11}}{\partial x^{2}} & \;={{\left[\frac{r^{2}+{\left(x^{\prime }-x\right)}^{2}}{r}]\right|}_{a_{2}}^{a_{1}}|}_{b_{2}}^{b_{1}}+x\frac{\partial^{2}{\text{A}}^{01}}{\partial x^{2}}+2\frac{\partial {\text{A}}^{01}}{\partial x}+y\frac{\partial^{2}{\text{A}}^{10}}{\partial x^{2}}-xy\frac{\partial^{2}{\text{A}}^{00}}{\partial x^{2}}-2y\frac{\partial {\text{A}}^{00}}{\partial x}\\ \frac{\partial {\text{A}}^{11}}{\partial y} & \;={{\left[-(y^{\prime }-y)r]\right|}_{a_{2}}^{a_{1}}|}_{b_{2}}^{b_{1}}+y\frac{\partial {\text{A}}^{10}}{\partial y}+{\text{A}}^{10}+x\frac{\partial {\text{A}}^{01}}{\partial y}-xy\frac{\partial {\text{A}}^{00}}{\partial y}-x{\text{A}}^{00}\\ \frac{\partial^{2}{\text{A}}^{11}}{\partial y^{2}} & \;={{\left[\frac{r^{2}+{\left(y^{\prime }-y\right)}^{2}}{r}]\right|}_{a_{2}}^{a_{1}}|}_{b_{2}}^{b_{1}}+x\frac{\partial^{2}{\text{A}}^{01}}{\partial y^{2}}+y\frac{\partial^{2}{\text{A}}^{10}}{\partial y^{2}}+2\frac{\partial {\text{A}}^{10}}{\partial y}-xy\frac{\partial^{2}{\text{A}}^{00}}{\partial y^{2}}-2x\frac{\partial {\text{A}}^{00}}{\partial y}\\ \frac{\partial {\text{A}}^{11}}{\partial z} & \;={{\left[zr]\right|}_{a_{2}}^{a_{1}}|}_{b_{2}}^{b_{1}}+x\frac{\partial {\text{A}}^{01}}{\partial z}+y\frac{\partial {\text{A}}^{10}}{\partial z}-xy\frac{\partial {\text{A}}^{00}}{\partial z}\\ \frac{\partial^{2}{\text{A}}^{11}}{\partial z^{2}} & \;={{\left[\frac{r^{2}+z^{2}}{r}]\right|}_{a_{2}}^{a_{1}}\;|}_{b_{2}}^{b_{1}}+x\frac{\partial^{2}{\text{A}}^{01}}{\partial z^{2}}+y\frac{\partial^{2}{\text{A}}^{10}}{\partial z^{2}}-xy\frac{\partial^{2}{\text{A}}^{00}}{\partial z^{2}}\\ \frac{\partial^{2}{\text{A}}^{11}}{\partial x\partial z} & \;={{\left[-\frac{(x^{\prime }-x)z}{r}]\right|}_{a_{2}}^{a_{1}}|}_{b_{2}}^{b_{1}}+x\frac{\partial^{2}{\text{A}}^{01}}{\partial x\partial z}+\frac{\partial {\text{A}}^{01}}{\partial z}+y\frac{\partial^{2}{\text{A}}^{10}}{\partial x\partial z}-xy\frac{\partial^{2}{\text{A}}^{00}}{\partial x\partial z}-y\frac{\partial {\text{A}}^{00}}{\partial z}\\ \frac{\partial^{2}{\text{A}}^{11}}{\partial y\partial z} & \;={{\left[-\frac{(y^{\prime }-y)z}{r}]\right|}_{a_{2}}^{a_{1}}|}_{b_{2}}^{b_{1}}+x\frac{\partial^{2}{\text{A}}^{01}}{\partial y\partial z}+y\frac{\partial^{2}{\text{A}}^{10}}{\partial y\partial z}+\frac{\partial {\text{A}}^{10}}{\partial z}-xy\frac{\partial^{2}{\text{A}}^{00}}{\partial y\partial z}-x\frac{\partial {\text{A}}^{00}}{\partial z}\\ \frac{\partial^{2}{\text{A}}^{11}}{\partial x\partial y} & \;={{\left[\frac{\left(x^{\prime }-x)(y^{\prime }-y\right)}{r}]\right|}_{a_{2}}^{a_{1}}|}_{b_{2}}^{b_{1}}+y\frac{\partial^{2}{\text{A}}^{10}}{\partial x\partial y}+\frac{\partial {\text{A}}^{10}}{\partial x}+x\frac{\partial^{2}{\text{A}}^{01}}{\partial x\partial y}+\frac{\partial {\text{A}}^{01}}{\partial y}-\cdots \\ & \;xy\frac{\partial^{2}{\text{A}}^{00}}{\partial x\partial y}-x\frac{\partial {\text{A}}^{00}}{\partial x}-y\frac{\partial {\text{A}}^{00}}{\partial y}-{\text{A}}^{00}\\ \frac{\partial {\text{B}}^{11}}{\partial x} & \;={{\left[-\frac{1}{2}(x^{\prime }-x)(zr+({\left(x^{\prime }-x\right)}^{2}+{\left(y^{\prime }-y\right)}^{2})\log(z+r))]\right|}_{a_{2}}^{a_{1}}|}_{b_{2}}^{b_{1}}+y\frac{\partial {\text{B}}^{10}}{\partial x}+x\frac{\partial {\text{B}}^{01}}{\partial x}+{\text{B}}^{01}-xy\frac{\partial {\text{B}}^{00}}{\partial x}-y{\text{B}}^{00}\\ \frac{\partial^{2}{\text{B}}^{11}}{\partial x^{2}} & \;={{\left[\frac{1}{2}zr+\frac{1}{2}(3{\left(x^{\prime }-x\right)}^{2}+{\left(y^{\prime }-y\right)}^{2})\log(z+r)]\right|}_{a_{2}}^{a_{1}}|}_{b_{2}}^{b_{1}}+x\frac{\partial^{2}{\text{B}}^{01}}{\partial x^{2}}+2\frac{\partial {\text{B}}^{01}}{\partial x}+\cdots \\ & \;y\frac{\partial^{2}{\text{B}}^{10}}{\partial x^{2}}-xy\frac{\partial^{2}{\text{B}}^{00}}{\partial x^{2}}-2y\frac{\partial {\text{B}}^{00}}{\partial x}\\ \frac{\partial {\text{B}}^{11}}{\partial y}= & {{\left[-\frac{1}{2}(y^{\prime }-y)\left(zr+\left({\left(x^{\prime }-x\right)}^{2}+{\left(y^{\prime }-y\right)}^{2}\right)\log\left(z+r\right)\right)]\right|}_{a_{2}}^{a_{1}}|}_{b_{2}}^{b_{1}}+y\frac{\partial {\text{B}}^{10}}{\partial y}+\cdots \\ & \;{\text{B}}^{10}+x\frac{\partial {\text{B}}^{01}}{\partial y}-xy\frac{\partial {\text{B}}^{00}}{\partial y}-x{\text{B}}^{00}\\ \frac{\partial^{2}{\text{B}}^{11}}{\partial y^{2}} & \;={{\left[\frac{1}{2}zr+\frac{1}{2}(3{\left(y^{\prime }-y\right)}^{2}+{\left(x^{\prime }-x\right)}^{2})\log(z+r)]\right|}_{a_{2}}^{a_{1}}|}_{b_{2}}^{b_{1}}+x\frac{\partial^{2}{\text{B}}^{01}}{\partial y^{2}}+y\frac{\partial^{2}{\text{B}}^{10}}{\partial y^{2}}+\cdots \\ & \;2\frac{\partial {\text{B}}^{10}}{\partial y}-xy\frac{\partial^{2}{\text{B}}^{00}}{\partial y^{2}}-2x\frac{\partial {\text{B}}^{00}}{\partial y}\\ \frac{\partial^{2}{\text{B}}^{11}}{\partial x\partial y} & \;=[(x^{\prime }-x)(y^{\prime }-y)\log(z+r)]{|}_{a_{2}}^{a_{1}}{|}_{b_{2}}^{b_{1}}+y\frac{\partial^{2}{\text{B}}^{10}}{\partial x\partial y}+\frac{\partial {\text{B}}^{10}}{\partial x}+x\frac{\partial^{2}{\text{B}}^{01}}{\partial x\partial y}+\cdots \\ & \;\frac{\partial {\text{B}}^{01}}{\partial y}-xy\frac{\partial^{2}{\text{B}}^{00}}{\partial x\partial y}-x\frac{\partial {\text{B}}^{00}}{\partial x}-y\frac{\partial {\text{B}}^{00}}{\partial y}-{\text{B}}^{00} \end{array}$$
