# Supplementary figures and images for: Application of low-order potential solutions to higher-order vertical traction boundary problems in an elastic half-space
Source: R Soc Open Sci. 2018 May 9;5(5):180203. doi: 10.1098/rsos.180203 (PMC5990738; doi:10.1098/rsos.180203)

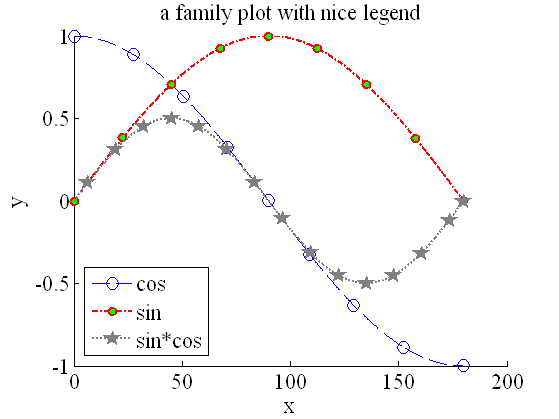

Supplement: Source codes and data files [file rsos180203supp1.zip › Source Code and Data for ESM/Strain Calculations/line_fewer_markers_v4/example.png]

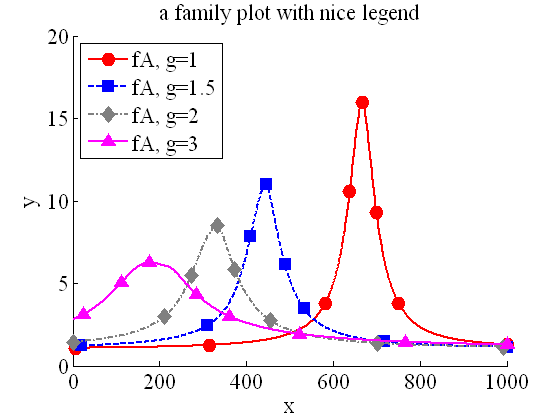

Supplement: Source codes and data files [file rsos180203supp1.zip › Source Code and Data for ESM/Strain Calculations/line_fewer_markers_v4/example_.png]
